# Transcriptome Analysis of *Populus euphratica* under Salt Treatment and *PeERF1* Gene Enhances Salt Tolerance in Transgenic *Populus alba* × *Populus glandulosa*

**DOI:** 10.3390/ijms23073727

**Published:** 2022-03-28

**Authors:** Xiao-Lan Ge, Lei Zhang, Jiu-Jun Du, Shuang-Shuang Wen, Guan-Zheng Qu, Jian-Jun Hu

**Affiliations:** 1 State Key Laboratory of Tree Genetics and Breeding, Research Institute of Forestry, Chinese Academy of Forestry, College of Forestry, Northeast Forestry University, Harbin 150040, China; gedalan@126.com (X.-L.G.); zhang_lei1142@163.com (L.Z.); dujiujun0517@163.com (J.-J.D.); 15501131025@163.com (S.-S.W.); 2 Collaborative Innovation Center of Sustainable Forestry in Southern China, Nanjing Forestry University, Nanjing 210037, China

**Keywords:** *Populus euphratica*, salt stress, transcriptome, WGCNA, *PeERF1*

## Abstract

*Populus euphratica* is mainly distributed in desert environments with dry and hot climate in summer and cold in winter. Compared with other poplars, *P. euphratica* is more resistant to salt stress. It is critical to investigate the transcriptome and molecular basis of salt tolerance in order to uncover stress-related genes. In this study, salt-tolerant treatment of *P. euphratica* resulted in an increase in osmo-regulatory substances and recovery of antioxidant enzymes. To improve the mining efficiency of candidate genes, the analysis combining both the transcriptome WGCNA and the former GWAS results was selected, and a range of key regulatory factors with salt resistance were found. The *PeERF1* gene was highly connected in the turquoise modules with significant differences in salt stress traits, and the expression levels were significantly different in each treatment. For further functional verification of *PeERF1*, we obtained stable overexpression and dominant suppression transgenic lines by transforming into *Populus alba × Populus* *glandulosa*. The growth and physiological characteristics of the *PeERF1* overexpressed plants were better than that of the wild type under salt stress. Transcriptome analysis of leaves of transgenic lines and WT revealed that highly enriched GO terms in DEGs were associated with stress responses, including abiotic stimuli responses, chemical responses, and oxidative stress responses. The result is helpful for in-depth analysis of the salt tolerance mechanism of poplar. This work provides important genes for poplar breeding with salt tolerance.

## 1. Introduction

Soil salinity is considered to be a principal problem for international agricultural manufacturing and sustainability [1]. Salt stress now severely limits the growth, development, yield, and quality of plants worldwide [2]. Salt stress is projected to affect more than 6% of the world’s land surface and over 20% of irrigated land, restricting the productivity and geographic dispersion of farmed crops.

For adaptation to saline environments, plants have evolved intricate methods for sensing environmental cues and coordinating metabolic processes and morphological characteristics through gene expression regulation [3]. Certain physiological structures of plants undergo adaptive changes to cope with unfavorable survival conditions, and such physiological changes are commonly seen in saline plants, where the outer skin cell walls thicken and leave flesh out to conserve water and accumulate salt [4]. Significant research has also been conducted around the changes in gene expression that occur in response to salt stress [2,5]. Salt tolerance genes in plants are associated with many pathways, such as phytohormone signaling, secondary metabolism, reactive oxygen species, and transcriptional regulation [6,7]. These genes can be classified as functional or regulatory. Some salt stress genes significantly enhance salt tolerance in transgenic plants; however, there are significant differences in the transcriptional response to salt tolerance in different plant species [8].

Compared with linkage analysis, genome-wide association study (GWAS) generally uses existing natural populations as materials, and does not need to construct specialized mapping populations. It has the advantages of being less time-consuming, having a large scope, and offering high accuracy, while weighted correlation network analysis (WGCNA) pays more attention to the mining and presentation of gene expression forms in different samples. The combined analysis of the two methods significantly improves the mining efficiency of candidate genes and provides a shortcut for GWAS and WGCNA to solve their limitations [9,10]. GWAS analysis of the seed germination rates of four different branches of *P. euphratica* in Northwest China showed that 38 single nucleotide polymorphisms (SNPs)were associated with seed salt tolerance, located within or near 82 genes [11]. To date, RNA sequencing has been widely used in various fields of life sciences [12,13]. Among them, comparative transcriptomics is widely used to identify differences in transcript abundance between varieties, organs and treatment conditions [14]. To identify genes exhibiting transcriptional changes, transcripts from poplars under different salt stress treatments and times were compared and analyzed with those from normally treated poplars [8].

The ethylene response factor (ERF) is a plant-specific transcription factor that plays a critical role in biotic and abiotic stresses. It regulates the expression of downstream genes by binding to cis-acting elements such as GCC-box/DRE/TGG in the promoter region of downstream genes through the AP2 structural domain, which in turn is involved in plant stresses such as adversity [15]. For example, the *ERF1* gene directly up-regulates A1SA by binding to its promoter, which affects auxin accumulation and plays a key role in inhibiting ethylene-induced primary root growth in *Arabidopsis thaliana* [16]. The tomato gene *JERF1* regulates the expression of ABA biosynthesis-related genes at the transcriptional level by binding to multiple cis-acting elements to improve tolerance to high salt and low temperature in tobacco [17].

The rapid propagation of transgenic plants has brought enormous agricultural and economic benefits to human beings [18,19]. However, there are also potential risks of endangering the survival of wild and non-target species, increasing resistance of target pests, causing genetic contamination and genetic drift. In order to solve the safety problems of genetically modified organisms (GMOs), measures such as international legislation, regulatory system, improving the labeling of GMOs, and strengthening international cooperation on the safety of GMOs have been implemented [20,21,22,23,24].

Poplar grows fast, has a long-life cycle, has strong adaptability and excellent material, and is widely used in greening, afforestation, and other aspects [25]. *P. euphratica* is mainly distributed in the northwest desert and other desertified areas of China, and is known for its extremely high tolerance to drought and salt stress. Compared to other poplar plants, *P.* *euphratica* is more resilient, and can grow in the presence of 200 mM NaCl, and even survives in 400 mM NaCl and mannitol [26,27]. *P. euphratica*, as a dominant tree species in desert ecosystems, has very important economic and ecological value, and is also an ideal model material for studying abiotic stress in woody plants [28,29,30,31,32,33,34]. There are numerous reports on the physiological characteristics of salt stress tolerance in *P. euphratica*, but studies on the molecular mechanisms of salt resistance are limited. This study aimed to investigate the response mechanisms of poplar to salt stress and to identify the key regulatory genes. In order to achieve this goal, physiological responses to salt stress were analyzed. The differentially expressed genes (DEGs) in young leaves of poplar were screened by RNA sequencing at four timepoints with two salt treatments. Gene Ontology (GO) and Kyoto Encyclopedia of Genes and Genomes (KEGG) analyses were performed on DEG to identify the different metabolic pathways involved in the salt stress response. We compared the overlapped genes between WGCNA modules and former GWAS results, and found that some genes, such as POPULUS_EUPHRATICA_07301 (*ERF1*), POPULUS_EUPHRATICA_13633 *(NAC21/22*), POPULUS_EUPHRATICA_28205 (*ITN1*), etc., are involved in the osmotic stress response of plants. The *PeERF1* gene was selected to transform with poplar for functional validation. RNA sequencing of *PeERF1* transgenic *P. alba × P. glandulosa* screened many stress-related genes and found that overexpressed transgenic *P. alba × P. glandulosa* functioned mainly after salt stress. Our results can help to screen excellent resistance gene resources in poplar, and combined with genetic engineering, it is important to breed resistant and high-quality tree species suitable for our needs.

## 2. Results

### 2.1. Validation of Salt Stress Treatment in P. euphratica

To explore the salt tolerance mechanism of *P. euphratica*, Plants at four-time points (0, 12, 24, and 48 h) and two treatment concentrations (A, 150 mM NaCl; B, 300 mM NaCl) under NaCl were employed in physiological research. Before the harvest season, different treatments were started at the same time, and all samples were collected at the same time [35]. To evaluate the physiological responses to salt stress, we assessed superoxide dismutase (SOD), peroxidase (POD), and malondialdehyde (MDA).

**Figure 1:**
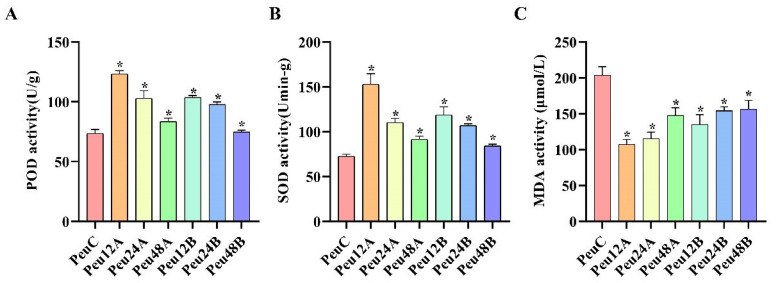
Physiological response to salt stress in the young leaves of *P. euphratica*. (**A**–**C**) Comparison of peroxidase (POD), superoxide dismutase (SOD), and malonaldehyde (MDA) contents between t different times and concentrations in *P. euphratica*; the control is water. Three biological replicates and three technical replicates were used in the studies. The student’s *t*-test was used to assess the data. *p* < 0.05 of three-time intervals (12, 24, and 48 h) versus the control (0 h) and two treatment concentrations (150 mM and 300 mM) versus the control (0 mM), respectively. Abbreviations: PeuC, *P. euphratica* control; A, 150 mM NaCl salt-stressed; B, 300 mM NaCl salt-stressed; 12, salt stress for 12 h; 24, salt stress for 24 h; 48, salt stress for 48 h (* *p*-value < 0.05).

Peroxidase is an important endogenous scavenger of reactive oxygen species, which is closely related to plant stress resistance. POD can reduce plant damage and remove excessive reactive oxygen species. Under 150 mM and 300 mM treatments, there was a significant upward trend from 0 h to 12 h and a significant decrease from 12 h to 48 h (Figure 1). Interestingly, SOD increased first and then decreased under salt stress. MDA decreased first and then increased at 150 mM and 300 mM.

### 2.2. Analysis of RNA Sequences and Identification of Differentially Expressed Genes in P. euphratica under Salt Stress

To further elucidate the molecular basis of salt response in *P. euphratica*, RNA sequencing (RNA-seq) was used for transcriptome analysis. The cDNA libraries were created separately from the leaves taken at two concentrations (150 mM and 300 mM) and four-time points (0 h, 12 h, 24 h, and 48 h) following salt stress, with three duplicates at each time point, and then sequenced on the Illumina Hiseq 2500 platform. After the initial sequencing readings have been checked for quality, 915,771,706 clean readings (137.37 Gb) were retained, and the paired-end was 125 bp for further analysis. The content of GC was 43.96%, and that of Q30 was 93.7% (Supplementary Table S1).

The FPKM values were determined based on the uniquely mapped reads for both control and salt stress conditions to determine the relative expression levels of genes. Using comparison analysis, the data of RNA-seq revealed a total of 18,002 differently expressed genes in reaction to salt stress. Out of the total DEGs, 8624 (47.9%) of those DEGs were up-regulated, while 9378 (52.1%) of them were down-regulated (Supplementary Table S2). A larger amount of variation in gene expression in response to salt stress are indicated by a broader dispersion.

**Figure 2:**
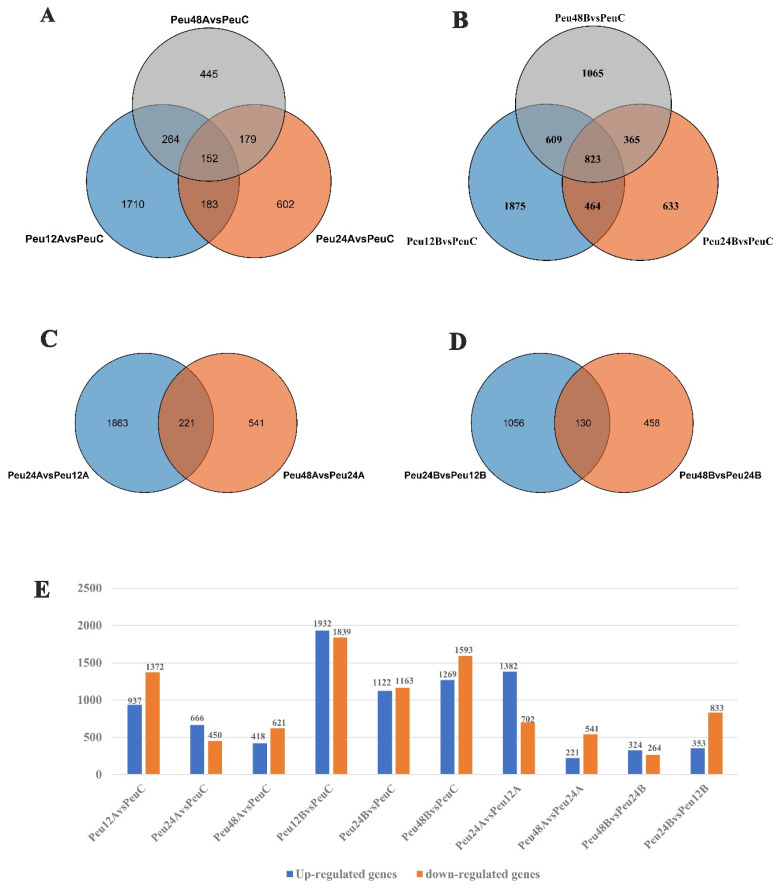
Statistics of DEGs of *P. euphratica* under Salt Stress. (**A**) Group I: Overlap of comparison Peu12A vs. PeuC, Peu24A vs. PeuC, and Peu48A vs. PeuC of up-regulated or down-regulated genes after NaCl treatment. (**B**) Group II: Overlap of comparison Peu12B vs. PeuC, Peu24B vs. PeuC, and Peu48B vs. PeuC of DEGs after 300 mM NaCl treatment. (**C**) Group III: Overlap of comparison Peu24A vs. Peu12A and Peu48A vs. Peu24A of DEGs after NaCl treatment. (**D**) Group IV: Overlap of comparison Peu24B vs. Peu12B and Peu48B vs. Peu24B of DEGs after NaCl treatment. (**E**) The number of up-regulated and down-regulated genes in all comparison combinations. Abbreviations: PeuC, *P. euphratica* control; A, 150 mM NaCl salt-stressed; B, 300 mM NaCl salt-stressed; 12, salt stress for 12 h; 24, salt stress for 24 h; 48, salt stress for 48 h.

DEGs were discovered in several comparisons and grouped into four groups: Group I for various treatment timepoints under 150 mM salt stress compare to control (Peu12A vs. PeuC, Peu24A vs. PeuC, and Peu48A vs. PeuC) (Figure 2A), Group II for different treatment timepoints under 300 mM salt stress compare to control (Peu12B vs. PeuC, Peu24B vs. PeuC, and Peu48B vs. PeuC) (Figure 2B), Group III for comparison with 150 mM salt stress at different times (Peu24A vs. Peu12A and Peu48A vs. Peu24A) (Figure 2C) and Group IV for comparison with 300 mM salt stress at different times (Peu24B vs. Peu12B and Peu48B vs. Peu24B) (Figure 2D). In the comparison of “Peu12B vs. PeuC,” (a total of 3771 DEGs including 1932 up-regulated genes and 1839 down-regulated genes) the biggest DEG set was discovered, which indicated that the young leaves of *P. euphratica* rapidly reprogrammed the cellular response at the transcriptome level at salt stress for 12 h. The lowest DEG set was discovered in the comparison “Peu48B vs. Peu24B” (588 DEGs including 324 up-regulated genes and 264 down-regulated genes), followed by comparison “Peu48A vs. Peu24A” (762 DEGs including 221 up-regulated genes and 541 down-regulated genes) (Figure 2E). In three comparisons, the overlapped DEGs of Group II (823 common genes, 4.57% of total DEGs) were bigger than those of the other groups, according to comparative analysis (Figure 2A,D). The most extensive subset of particular DEGs was “Peu12B vs. Peu12A” (6086 DEGs in Group III) and “Peu12A vs. PeuC” (3771 DEGs in Group II). Furthermore, in the fourth group, there were few overlapping of differential genes (Figure 2D).

### 2.3. GO and KEGG Enrichment Analysis of DEGs

The identified DEGs were annotated with 18 biological processes (BP), 9 cellular components (CC), and 9 molecular functions (MF) in GO categories (Supplementary Figure S1), and significantly enriched (*p* ≤ 0.001) into 122 GO terms (Supplementary Table S3). The terms “cellular process”, “metabolic process”, “single-organism process”, “biological regulation”, “localization”, “regulation of biological process” and “response to stimulus”, were the dominant groups in the biological processes; “cell”, “cell part”, “membrane”, “membrane-part”, “organelle”, and “macr-molecular complex” were of the representative groups in the cellular components. Among the molecular functions, a great number of DEGs were focused on categories of “binding”, “catalytic activity”, and “transporter activity”.

To elucidate the molecular pathways driving salt tolerance in *P. euphratica*, the DEGs were characterized using the GO knowledgebase (http://geneontology.org/ (accessed on 20 December 2020)). The enhanced functions for all DEGs owing to salt stress are shown in GO enrichment scatterplots (Supplementary Figure S2 and Supplementary Table S4). DEGs were enriched including “cellular carbohydrate metabolic process” and “photosynthesis” that were prominent in Group I comparisons (Peu12A vs. PeuC, Peu24A vs. PeuC and Peu48A vs. PeuC), but did not enrich in Group II comparisons (Peu12B vs. PeuC, Peu24B vs. PeuC and Peu48B vs. PeuC). “Cell wall organization or biogenesis” and “aminoglycan catabolic process” were enriched in Group III comparisons (Peu24A vs. Peu12A and Peu48A vs. Peu24A), but did not enrich in Group IV (Peu24B vs. Peu12B and Peu48B vs. Peu24B). GO terms under the MF category include “carboxylic ester hydrolase activity” was enriched in Group I, “heme binding” and “tetrapyrrole binding” were enriched in Group II. In the CC category, DEGs were significantly enriched in GO terms of “extracellular region”, “external encapsulating structure”, “cell wall”, “cell periphery”, and “apoplast” in all groups.

To further understand the molecular response to salt of *P. euphratica* at the pathway level, we used KEGG pathways method to perform enrichment analysis. In this approach, a total of 20 routes were greatly enhanced (Supplementary Figure S3 and Supplementary Table S5). In particular, “Plant hormone signal transduction”, “Galactose metabolism”, “MAPK signaling pathway-plant”, and “Cutin, suberine and wax biosynthesis” were significantly enriched in most comparisons. The pathway of “Phenylpropanoid biosynthesis” was highly enriched in the early and middle phases (Peu24A vs. PeuC and Peu24A vs. Peu12A); the pathway of “Cysteine” and “Amino sugar and nucleotide sugar metabolism” was significantly enriched in medium and late stages (Peu48B vs. PeuC and Peu48A vs. Peu24A). Moreover, the pathways of “Starch and sucrose metabolism”, “Nitrogen metabolism’ and “Flavonoid biosynthesis” were specifically enriched in different stages in Group II and Group III comparisons.

### 2.4. Identification of WGCNA Modules Related to Phenotypic Traits

**Figure 3:**
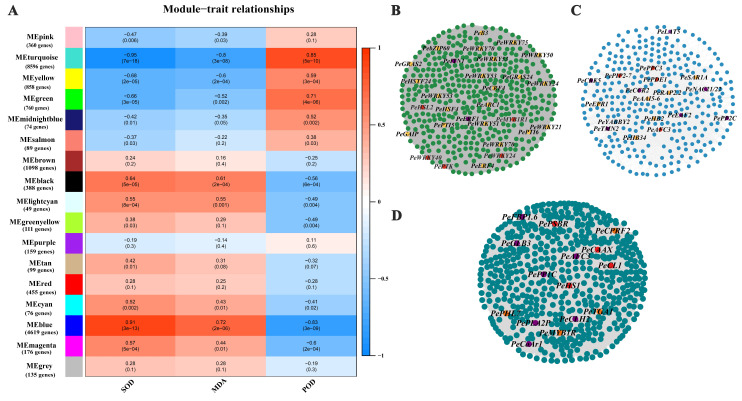
Weighted gene co-expression network analysis of DEGs. (**A**) Module–trait relationships. Correlations between module–trait weights and their accompanying *p*-values (in parenthesis). The 17 modules are depicted in the left panel, along with the number of genes in each module. The color scale on the right displays module–trait correlation from −1 (blue) to 1 (red). (**B**) Cytoscape depiction of co-expressed genes in module–trait turquoise. Yellow and red nodes represent transcription factors and high connectivity genes, respectively. Purple represents genes that overlap with GWAS results. (**C**) Cytoscape representation of co-expressed genes in module–trait green. (**D**) Cytoscape representation of co-expressed genes in module–trait blue.

We used WGCNA to construct a co-expression network to study the interrelationships among salt responsive genes. The DEGs state that their expression patterns of expression were split into seventeen distinct modules. Seventeen separate modules were recognized and labeled with various colors in a dendrogram (Figure 3A). These results indicated that different degrees of salt stress development were under phenotypic traits control. The turquoise module of 9438 DEGs, green of 763 DEGs and blue of 4624 DEGs were significantly and stably correlated with phenotypic traits including POD, SOD, and MDA (r > 0.8 or r < −0.8). The correlation network of the turquoise, green and blue module genes with WGCNA edge weight >0.3 were shown in Figure 3B–D.

After analyzing the overlapped genes between WGCNA modules and prior GWAS results [11], we found 23, 7, and 2 candidate genes for GWAS intervals in the turquoise, blue and green modules, respectively (Supplementary Table S6). Analysis of major transcription factor (TF) families presented in different modules revealed that they were differentially sensitive to salt response effects (Supplementary Figure S6). Overall, TFs were strongly represented in turquoise module (217 TFs, 46.5% of DEGs), which including 55 MYB, 26 AP2, 22 b-ZIP, 18 B3, 17 WRKY, 15 ARF, 15 GRAS, 11 GATA, 10 TCP, 7 SBP, 7 FAR1, 6 HSF, 3 E2F/DP, 2 SAP, 2 ZF-HD, and 1 HD-ZIP (Supplementary Table S7). We observed that some genes (*ERF1*, *NAC21/22*, *MYB48*, *WRKY6* etc.) are also involved in plants’ osmotic stress response. The ethylene response factor (ERF) family is one of the largest plant-specific transcription factor families, playing an important role. Among them, the *PeERF1* gene was highly connected in the turquoise module, and the traits were significantly different, and the expression levels of each treatment stage were significantly different. We selected *PeERF1* for functional validation of transgenic poplar.

**Figure 4:**
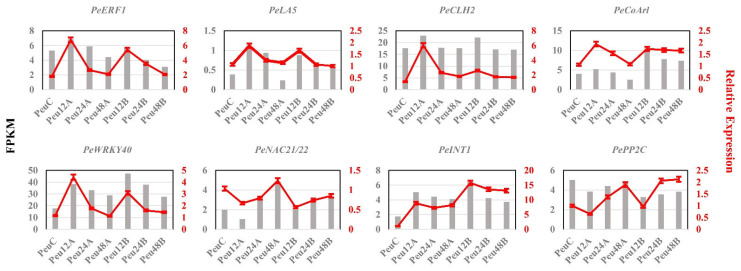
RT-PCR was used to validate the expression of eight genes. The left gray histogram represents the qRT-PCR results. The red broken line on the right represents the FPKM. Values are presented as means ± SD of three independent measurements.

For quantitative Real-Time PCR (qRT-PCR) validation, eight genes were chosen from the DEG list (Supplementary Table S8), which include Ethylene-responsive transcription factor 1B (*PeERF1*, POPULUS_EUPHRATICA_07301), Probable protein phosphatase 2C (*PePP2C*, POPULUS_EUPHRATICA_06665), Lysophosphatidyl acyltransferase 5 (*PeLA5*, POPULUS_EUPHRATICA_35413), WRKY DNA-binding protein 40 (*PeWRKY40*, POPULUS_EUPHRATICA_12662), NAC domain-containing protein 21/22 (*PeNAC21/22*, POPULUS_EUPHRATICA_13633), Ankyrin repeat-containing protein ITN1 (*PeITN1*, POPULUS_EUPHRATICA_28205), 3-hydroxy-3-methylglutaryl-coenzyme A reductase 1 (*PeCoAr1*, POPULUS_EUPHRATICA_32184) and Chlorophyllase-2(*PeCLH2*, POPULUS_EUPHRATICA_20786). The expression patterns of the eight genes revealed a significant rise at 12 h, followed by a slow fall but still greater than the control at 24 h and 48 h (Figure 4). This pattern of expression supported the accuracy of the RNA-Seq data.

### 2.5. Cloning of PeERF1 and Protein Sequence Comparisons

To verify the reliability of WGCNA, we selected *PeERF1* to conduct functional verification. *P. euphratica* provided the cDNA fragment for *PeERF1*. It comprises a 663 bp coding sequence that encodes 221 amino acids with a molecular weight of 53.814 k Da (Supplementary Figure S5).

To investigate the evolutionary relationship of PeERF1, we performed multiple sequence alignment and evolutionary analysis of ERF1 amino acid sequences using Bioedit and MEGA5.0 software. We found that the ERF protein sequences of 10 plants had conserved the AP2 domain (Supplementary Figure S6). The amino acid sequence consistency was 82–98%. Among them, ERF1 protein has high homology with ERF1 protein of *Populus deltoides* (H0E87_010489), *Populus alba* (XP_034925586.1), *Salix suchowensis* (KAG5240668.1), and *P. trichocarpa* (XM_024607193.1).

### 2.6. PeERF1 Subcellular Location and Transcriptional Activity Analysis

To explore the distribution of *PeERF1* transcription factor expression products in cells, we constructed expression vectors containing transcription factors using the GFP fusion protein reporter, and transiently introduced the successfully constructed expression vectors into *Nicotiana tabacum* leaves [36]. As shown, the fluorescent signal of the 35S: *PeERF1*-YFP protein was exclusively observed in the nucleus. These results show that *PeERF1* is a nuclear gene localized (Supplementary Figure S7A,B).

**Figure 5:**
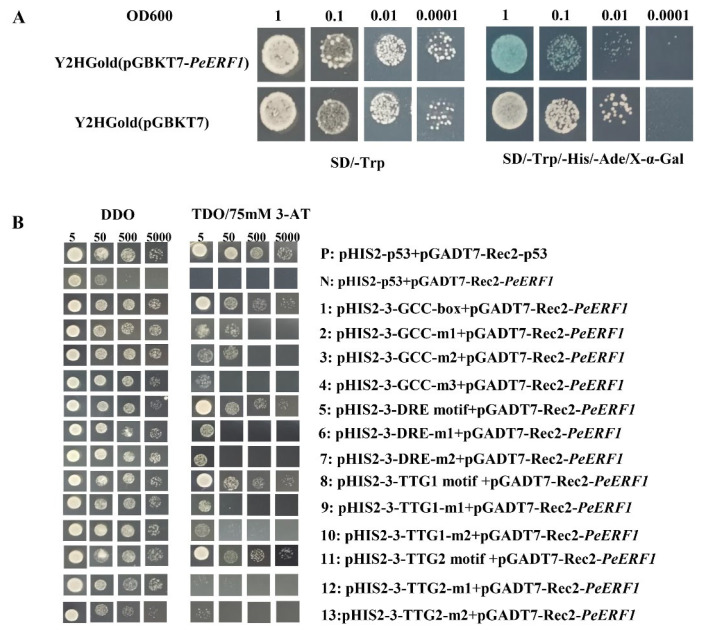
Self-activation experiment and Y1H analysis of *PeERF1*. (**A**) As a negative control, yeast cells transformed with the empty pGBKT7 vector were employed. As a positive control, pGADT7-T co-transformed yeast cells were employed. (**B**) *PeERF1* binding to the GCC-box, DRE, and TTG motif was studied using Y1H. DDO (Double Dropout Medium): SD medium devoid of leucine (Leu) and tryptophan (Trp), TDO (Triple Dropout Medium): SD medium devoid of leucine (Leu), tryptophan (Trp), and histidine (His). 1, 0.1, 0.01, 0.001: each spot, yeast cells were serially diluted 1, 10, 100, or 1000 times. P: Positive control, p53HIS2 + pGADT7-Rec2-53, N: negative control, p53HIS2 + pGADT7-*PeERF1*; 1, 5, 8 and 11: *PeERF1* binding to GCC-box, DRE motif, TGG1 and TGG2 core sequence; 2–4, 6–7, 9–10, 12–13: *PeERF1* binding to the GCC-box, DRE, TGG1 and TGG2 motif mutations.

To investigate *PeERF1* transcriptional activity, we created the pGBKT7-*PeERF1* fusion vector and converted into Y2HGold yeast cells. Negative control yeast cells were Pgadt7 co-transformed yeast cells. Positive control yeast cells were pGADT7-T co-transformed yeast cells. As shown in Figure 5A, it has self-activating activity in yeast. 

### 2.7. Promoter Activity and Relevant Transient Acting Elements Analysis of PeERF1

To evaluate the tissue-specific expression of the *PeERF1* gene, various DNA segments of 1524 bp from the promoter of the *PeERF1* promoter were inserted into poplar genome to induce the production of the β-glucuronidase (GUS). GUS histochemical staining revealed that GUS was expressed mostly in the top young leaves of poplar, but not in the stems or roots (Supplementary Figure S7).

In order to investigate the role of *PeERF1* gene and elements, the GCC box, DRE motif, TTG1 motif, and TTG2 motif, which can bind to ERF subfamily proteins, were selected with reference to the relevant literature [32,37,38,39]. To determine whether PeERF1 can also bind to these elements, we performed a yeast one-hybrid assay. The constructed pHIS2 reporter vector containing the GCC-box, DRE motif, TTG1 motif, and TTG2 motif and the pGADT7-Rec2 effector vector carrying *PeERF1* and the pGADT7-Rec2 effector vector carrying *PeERF1* were co-transformed into Y187 yeast cells. The yeast cells of the two co-transformed reporter and effector vectors were able to grow on DDO and TDO/3-AT (75 mM) medium, demonstrating that *PeERF1* can bind to the GCC-box, DRE motif, TTG1 motif, and TTG2 motif, just as the positive control (Figure 5B).

### 2.8. Morphological Changes of Transgenic P. alba × P. glandulosa under Salt Stress

**Figure 6:**
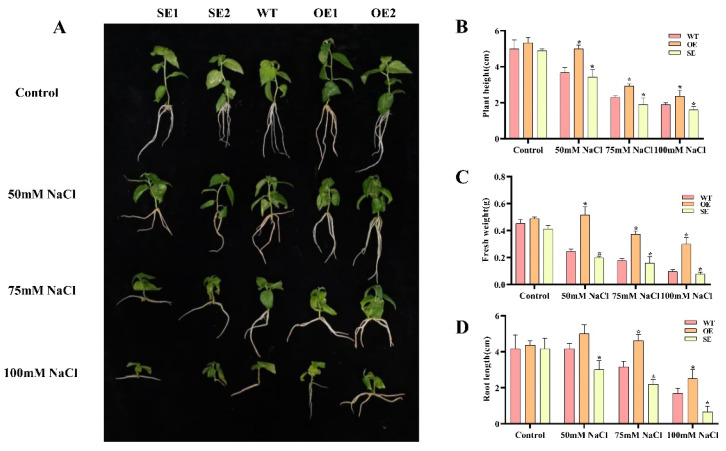
Transgenic *P. alba × P. glandulosa* morphological traits under salt stress. SE1, SE2, OE1, OE2: various transgenic poplar lines; WT, wild type poplar. (**A**–**D**) 1-month-old *P. alba × P. glandulosa* phenotypes on 0, 50, 75, and 100 mM NaCl rooting media. Under salt stress, the height, root length, and fresh weight were measured in transgenic and WT. The standard deviation is shown by the error bar. The presence of an asterisk implies that there is a substantial difference between transgenic and WT (*t*-test, * *p* < 0.05).

We selected the high transformation efficiency transgenic *P. alba × P. glandulosa* healing culture method for transformation to obtain transgenic plants [40]. Four transgenic *P. alba × P. glandulosa* lines and the wild type (WT) were given different treatments of 0, 50, 75, and 100 mM NaCl. Under normal circumstances, the plant height, root length, and fresh weight of 35S: *PeERF1* transgenic poplar were 1.06, 1.05, and 1.08 times more than those of WT, respectively. 35S: SRDX-*PeERF1* transgenic poplar was 0.91, 0.99, and 0.91 times more than WT. Additionally, plant height, root length, and fresh weight of 35S: *PeERF1* transgenic poplars were 1.36, 1.2, and 2.1 times higher than WT under 50 mM NaCl conditions, respectively. 35S: SRDX-*PeERF1* transgenic poplar were 0.93, 0.72, and 0.81 times than that of WT, respectively. Under 75 mM NaCl conditions, the values of 35S: *PeERF1* transgenic poplar were 1.35, 1.45, 2.11 times, respectively. The values of 35S: SRDX-*PeERF1* transgenic poplar were 0.92, 0.69, 0.9 times, respectively. Additionally, under 100 mM NaCl conditions, the ratio of 35S:*PeERF1* transgenic poplar changed to 1.15, 1.47, 3.1 times compared to WT (Figure 6A–D). The ratio of *35S: SRDX-PeERF1* transgenic poplar changed to 0.64, 0.39, 0.8 times compared to WT.

**Figure 7:**
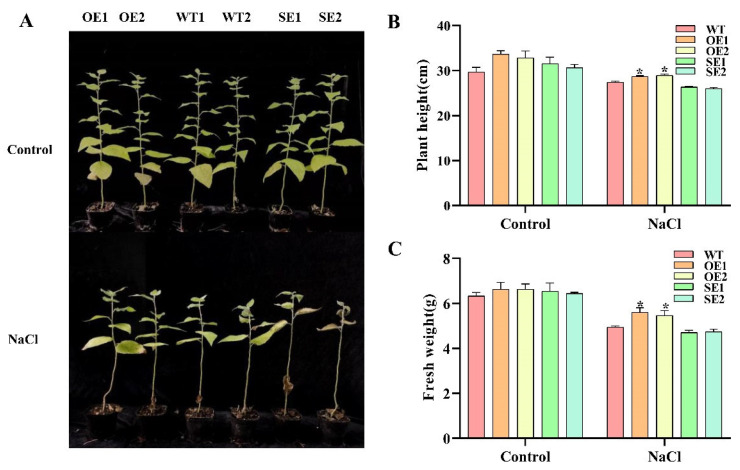
WT and 1-month-old transgenic *P. alba* × *P. glandulosa* were given a 7-day treatment with 150 mm salt. (**A**–**C**) Under salt stress, the growth state, height, and fresh weight were measured in transgenic and WT (* *p*-value < 0.05).

To compare transgenic and WT under salt stress, one-month-old plants were treated with 150 mM salt concentration for 7 days. Plant height and fresh weight were determined. Transgenic poplars and WT grew normally in the control environment without obvious symptoms. However, under salt stress, WT started to wilt slightly and leaves fell off then. The growth state of 35S:*PeERF1* (OE) was better than that of WT, while the growth state of 35S: SRDX-*PeERF1* (SE) transgenic lines was worse than that of WT (Figure 7A). The plant height and fresh weight of all OE plants were significantly higher than those of WT, while SE showed the opposite trend (Figure 7B,C).

### 2.9. Analysis of Physiological Characteristics of PeERF1 Transgenic P. alba × P. glandulosa

**Figure 8:**
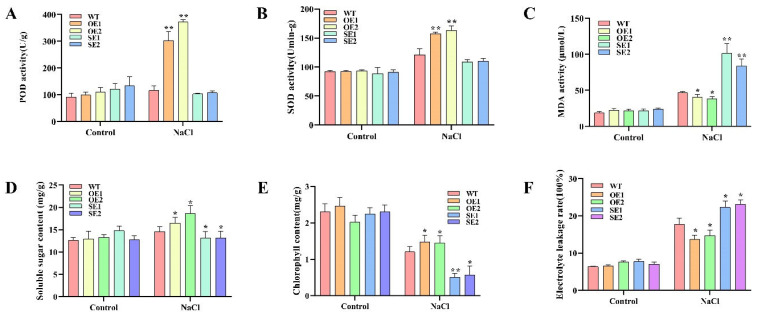
Analysis of physiology and gene expression in response to salt stress. OE5, OE7, OE11, SE4, SE6, SE7: different transgenic lines; WT, wild type. (**A**–**F**) POD, SOD, MDA, soluble sugar, chlorophyll, and relative conductivity levels were compared between transgenic and WT; the control is water (* *p*-value < 0.05; ** *p*-value < 0.01).

To investigate the role of *PeERF1* in osmotic stress, we measured physiological indicators of transgenic lines and WT under salt treatment. The accumulation of ROS in *PeERF1* transgenic is lower than in WT after the treatments. After salt treatment, the transgenic plants’ peroxidase (POD), soluble sugar content, and superoxide dismutase (SOD) levels were greater than the WT plants (Figure 8A,B,D), although the transgenic plants’ malondialdehyde (MDA) levels were lower, indicating that scavenging activity was increased (Figure 8C). All of these findings suggest that *PeERF1* overexpression improves salt tolerance.

The electrolyte leakage rate (LR) and leaf chlorophyll content (CC) were measured to assess the plants’ growth stage. The LR and CC of transgenic and WT lines were not substantially different under normal growing conditions (Figure 8E,F). Under salt stress, the OE lines grew faster than the WT, with considerably greater LR and CC than the WT. The SE lines grew in the opposite direction, with much lower LR and CC than the WT.

**Figure 9:**
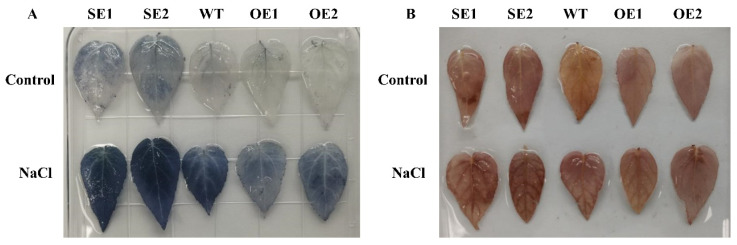
Staining with histochemical agents. (**A**) DAB staining of hydrogen peroxide. (**B**) NBT superoxide staining; WT, wild type; OE, transgenic poplar with 35S: *PeERF1* overexpression. SE, 35S: SRDX-*PeERF1* suppresses transgenic poplar expression.

The contents of hydrogen peroxide and superoxide were detected using Nitrotetrazolium blue chloride (NBT) and 3,3′-diaminobenzidine (DAB), respectively. Between transgenic and control lines, there was no obvious difference in NBT staining. After salinization, the staining area of 35S: *PeERF1* transgenic was substantially less than that of WT after salinization. DAB staining has a similar trend (Figure 9). The staining area of transgenic 35S: SRDX-*PeERF1* that suppressed expression was substantially bigger than that of WT. DAB staining has a similar trend.

### 2.10. Expression Analysis of Downstream Genes Regulated of PeERF1

**Figure 10:**
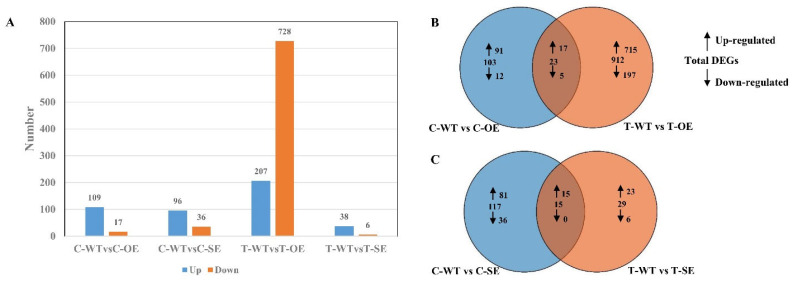
Statistics on gene expression differences between transgenic and WT before and after salt stress. (**A**) In all comparison combinations, the number of up-regulated and down-regulated genes. (**B**) Overlap of comparison C-WT vs. C-OE and T-WT vs. T-OE, of up-regulated or down-regulated genes after NaCl treatment. (**C**) Overlap of comparison C-WT vs. C-SE and T-WT vs. T-SE, of up-regulated or down-regulated genes after NaCl treatment.

To further investigate the regulation of those downstream genes to improve salt tolerance in plants. Based on the results of the analysis of variance, we identified genes as substantially different with FDR (*p*-value) < 0.05 and |log2fc| > 1. 126 differentially expressed genes were identified in the control from transgenic *P. alba × P. glandulosa* overexpressing the *PeERF1* gene, of which 109 genes were up-regulated and 17 genes were down-regulated (Figure 10). A total of 132 differentially expressed genes were identified in transgenic poplars, of which 96 genes were up-regulated and 36 genes were down-regulated. In the treatment group, 935 differentially expressed genes were identified from transgenic poplars overexpressing *PeERF1*, of which 207 genes were up-regulated and 728 genes were down-regulated. A total of 44 differentially expressed genes were identified in transgenic poplars, of which 38 genes were up-regulated and 6 genes were down-regulated.

**Figure 11:**
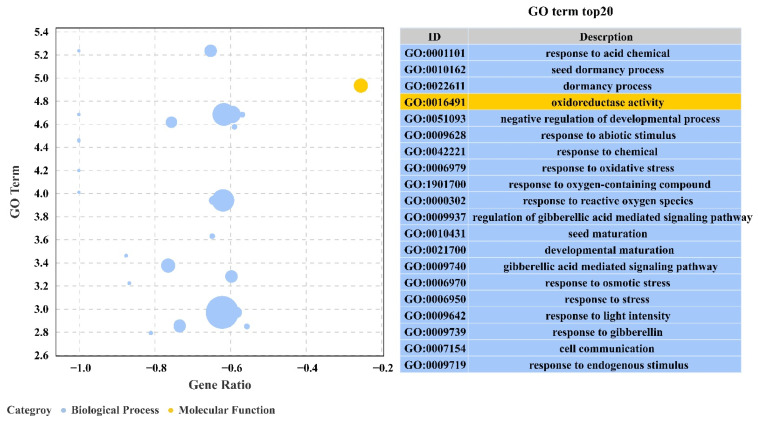
Analysis of GO enrichment of DEGs in the transcriptome under salt stress.

To further investigate the function of these genes, overexpressed and WT differential genes under salt stress were classified into three categories: biological processes, cellular components and molecular functions. These genes are associated with response to acidic chemicals, seed dormancy process, dormancy process, oxidoreductase activity, negative regulation of the developmental process, response to abiotic stimulus, response to chemical, response to oxidative stress, response to oxygen-containing compound, and response to reactive oxygen species (Figure 11).

Overexpression of transgenic *PeERF1* was compared between salt stress treatments and controls using a *q*-value of ≤0.05. There were 88 significantly enriched pathways with 621 differentially significant genes involved in the salt stress treatment. The top 20 pathways by the number of differential genes are shown in Figure 11. 

**Figure 12:**
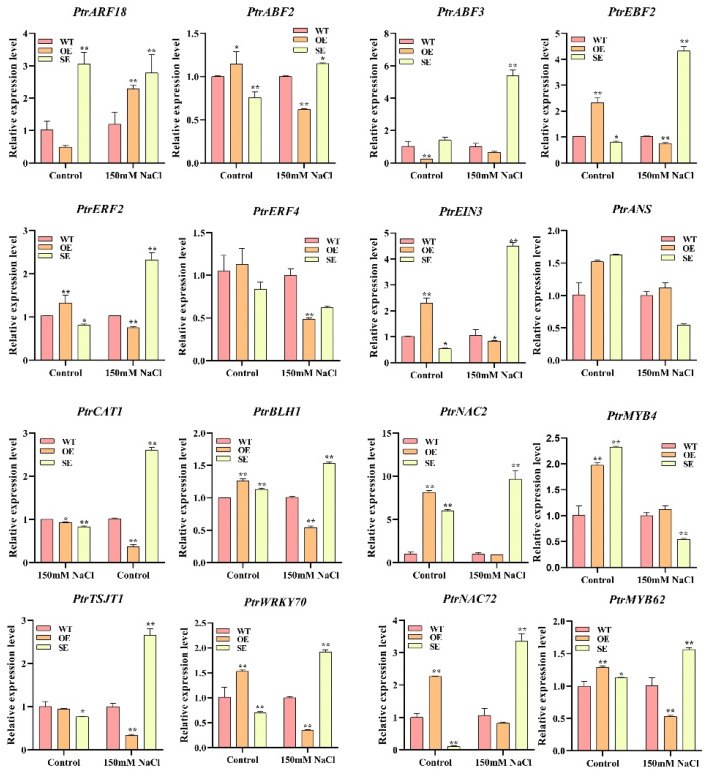
RT-qPCR verified the DEGs of RNA-seq (* *p*-value < 0.05; ** *p*-value < 0.01).

To verify the accuracy of the transcriptome sequencing results, we selected 16 stress-related genes, designed primers in their conserved domains for RT-PCR experiments, and then compared them with the transcriptome results. The results presented were nearly identical to the transcriptome sequencing results (Figure 12).

## 3. Discussion

The response signal pathway of abiotic stress is related to the physiological response of plants [41]. Treatment of *P. euphratica* with different concentrations of salt (50, 100, and 150 mM) reduced stomatal opening and leaf photosynthetic capacity, and the activities of POD and SOD were significantly higher than those of the control group. This is similar to the results of physiological indicators of *P. euphratica* treated at different times in our study (Figure 1). Some stress-related genes of *P. euphratica* have also been studied in plants. For example, Compared with the control, OE-*PeCBF4a* exhibited higher SOD activity and significantly reduced MDA content, indicating that transgenic OE-*PeCBF4a* poplars were more tolerant to salt stress [42]. The activities of antioxidant enzymes such as SOD, POD, and CAT in tobacco plants overexpressing *PeREM1.3* under salt stress significantly increased and decreased ROS levels, maintain ROS homeostasis, and improved the salt tolerance of plants [43]. In this study, the changes of POD, SOD, and MDA activities, soluble sugar content, chlorophyll content and relative electrical conductivity of transgenic poplar showed that the *PeERF1* gene could significantly improve its salt tolerance (Figure 9).

Based on the association analysis technology between genes and traits, WGCNA can reduce the dimensionality of complex data and simplify it into several modules. Weiss proposed Gene Module Association Study (GMAS) as a supplement to GWAS analysis results [44]. By integrating the results of GWAS and WGCNA analysis, Farber found that this method can significantly improve the mining efficiency of the micro-effect sites of the yellow seed phenotype, and found a shortcut for GWAS to solve its own limitations [45]. GWAS on the salt tolerance index of seed germination rate in four different branches of *P. euphratica* in Northwest China. A total of 38 SNP were found to be associated with seed salt tolerance, located within or near 82 genes [11]. Comparing the results with the GWAS, it was found that there were 41 overlapping genes in the 17 modules in WGCNA. qRT-PCR analysis of eight genes showed that the results were consistent with the transcriptome data (Figure 4). For example, *NAC21/22* responds to salt stress by indirectly regulating two metabolic pathways, phytohormone signaling and mitogen-activated protein kinase signaling [46]. The *AtITN1* mutant negatively regulates salt stress by mediating the *BOHC* and *RBOHD* genes [47].

In order to verify the efficiency and reliability of WGCNA data screening for salt tolerance genes, *PeERF1* gene was selected for salt tolerance analysis, and the positive response of *PeERF1* gene to plant salt stress was demonstrated by functional verification. In this study, we proved that PeERF1 is a nuclear localization protein with transcriptional autoactivation activity. Overexpression of *PeERF1* gene can enhance the salt tolerance of transgenic poplars, and dominant suppression of transgenic lines can inhibit the salt tolerance of transgenic plants. This result provides new progress and evidence for the involvement of *PeERF1* gene in the study of plant salt tolerance.

To obtain more information about molecular pathways underlying *PeERF1*’s response to salt stress, transcriptome sequencing of *PeERF1* (OE), SRDX-*PeERF1* (SE) strains and WT was carried out before and after salt stress treatments. In the analysis of GO enrichment of over differential genes after stress, it was found that most GOs were mainly associated with response to acid chemical, seed dormancy process, dormancy process, oxidoreductase activity, negative regulation of developmental process, response to abiotic stimulus, response to chemical, response to oxidative stress, response to oxygen-containing compound, response to reactive oxygen species and regulation of gibberellic acid mediated signaling pathway were found to be associated. The stress-related genes studied are as follows. *PtrERF2* plays an important role in the growth and stress resistance of plants among the homologous genes of other plants [48]. Overexpression of miR160 could cause degradation of the *ARF18* target gene transcript and increased salt tolerance in miR160 overexpress transgenic plants [49]. *PtrWRKY70* enhanced tolerance to osmotic stress among its homologous genes in other plants [50]. *PtrCAT1* belongs to the CAT enzyme family, which in other plant homologous genes can eliminate excess reactive oxygen species during stress resistance [51,52,53]. Thus, genes downstream of *PeERF1* may respond to abiotic stresses through different biological processes or pathways, thereby enhancing plant stress tolerance.

Except for *PeERF1*, which was functionally validated in our investigation, the other genes may also play a role in plant salt tolerance. For example, compared with wild type plants, *ZmPP2C-A1*, *ZmPP2C-A2*, and *ZmPP2C-A6* overexpression plants displayed greater germination rates after ABA and NaCl treatments than wild type plants in *A. thaliana* [54]. Although there have been no reports of *CLH2* function in salt tolerance, it has been confirmed that *AtPKL-1* mutant caused the up-regulation of chlorophyll degradation gene *CLH2* gene under cold stress in *A. thaliana* [55]. More research is needed to determine whether these genes contribute to salt tolerance in *P. euphratica*.

## 4. Materials and Methods

### 4.1. Plant Growth and NaCl Treatment

All materials used in this study were seedlings because the phenotype of poplar from seedling to mature stage is more prominent than that of older trees, and the seedling stage can eliminate environmental differences. Freshly matured seeds were harvested from the *P. euphratica* sown, germinated in the culturing room, and the seedlings were moved to tissue culture bottles for clone propagation. Under the aseptic condition, the growth of the plant was the same on the rooting medium. After the fibrous roots grew, they were transplanted into a pot culture containing peat soil and vermiculite (the ratio of peat soil and vermiculite was 3:1). Under the condition of long sunshine, it grows in a growth chamber underneath 26–28 °C, light for 16 h, and dark for 8 h. Plants have been subjected to 150 mM and 300 mM NaCl to simulate four-time variables of salt stress (0 h, 12 h, 24 h, and 48 h). At the cease of every time point, young leaves have been harvested, straight away frozen in liquid nitrogen, and saved in a −80 °C freezer for similar evaluation and sequencing. We made three biological replicates. (For the convenience of marking the following *P. euphratica* as Peu, 150 mM as A, and 300 mM as B).

### 4.2. Measurement of the Enzyme Activity of POD, MDA, and SOD

We selected 3-month-old plants and treated them with 150 mM NaCl and 300 mM NaCl for 12 h, 24 h, and 48 h. Plants of 0.5 g were harvested, crushed in liquid nitrogen, and enzymes were extracted with extraction buffer. Subsequently, the extraction was centrifuged at 12,000× *g* for 10 min, and the supernatant was used for the enzyme activity measurement. Cell superoxide dismutase (SOD), malondialdehyde (MDA), and peroxidase (POD) were measured (Solarbio, Beijing, China).

### 4.3. RNA Extraction and Illumina Deep Sequencing

A Plant Total RNA Extraction Kit was used to extract total RNA (TIANGEN, Beijing, China). The purity and integrity of RNA were checked by nanodrop, agarose gel electrophoresis, and Agilent Bioanalyzer 2100 system (Agilent Technologies Co., Ltd., Beijing, China). The Illumina Hiseq platform was used to sequence the library (LC Sciences, Houston, TX, USA), and a 150 bp double-terminal analysis used to be generated. Clean readings had been mapped to the reference *P. euphratica* version 2 (v 2.0) genome [56] the usage of HISAT2 [57]. 

### 4.4. Sequencing Data Processing and Analysis

Gene expression levels were calculated as reads per kilobase of transcript sequence per million base pairs sequenced (FPKM) using Cufflinks [58]. Differentially expressed genes (DEGs) were identified by DESeq [59]. A threshold of padj ≤ 0.05 was used to retrieve DEGs.

To investigate the biological function of DEGs, Blast2GO was used for gene set enrichment and Gene Ontology (GO) terminology [60]. The KEGG (Kyoto Encyclopedia of Genes and Genomes) tool was used to investigate metabolic pathways [61]. The co-expression network was examined using the R package WGCNA [62]. The graphical network created by Cytoscape 3.9.0 software [63].

### 4.5. qRT-PCR Analysis of Illumina Sequencing Results

We selected eight candidate DEGs for quantitative Real-Time PCR, which was used to confirm the accuracy of the RNA-Seq analysis. RNA was extracted and cDNA was reverse transcribed with HiScript qRT Super Mix (+gDNA wiper) (TIANGEN, Beijing, China). An amount of 1 µ cDNA was used as a template for quantitative Real-Time PCR (qRT-PCR), amplified using a Roche Light Cyclerreg 96 Real-Time PCR detection system and SYBR Premix Ex Taq™ (Takara, Dalian, China). The initial denaturation was performed at 95 °C for 5 min, followed by 35 denaturation cycles of 30 s at 95 °C, 30 s of annealing, and 20 s of extension at 72 °C. The primers were created using Primer Premier 5 software (PREMIER Biosoft, Palo Alto, CA, USA) and are listed in Supplementary Table S8. For each gene, three biological replicates were employed. The relative expression levels of the DEGs were calculated using the2^−∆∆ct^ method and normalized to the expression of the internal reference gene actin [64].

### 4.6. Sequence Comparisons and Phylogenetic Analysis

The sequence data for *PeERF1* were retrieved and downloaded from Genome Warehouse (https://ngdc.cncb.ac.cn/gwh/Assembly/865/show (accessed on 20 January 2021)) and NCBI (https://www.ncbi.nlm.nih.gov/ (accessed on 20 January 2021)). Amino acid sequence alignments of the *PeERF1* proteins were performed using the software Bioedit. A phylogenetic tree of *PeERF1* proteins based on the neighbor-joining method was constructed with MEGA5.1. The sequences of the genes that had the most similarities were obtained from the Phytozome (https://phytozome.jgi.doe.gov/pz/portal.html (accessed on 20 January 2021)) and Ensemble (https://plants.ensembl.org/index.html (accessed on 20 January 2021)) websites.

### 4.7. Plasmid Construction and P. alba × P. glandulosa Transformation

To test the functioning of the candidate genes, we used gene transformation in *P. alba × P. glandulosa*. The coding sequences of the candidate genes were cloned into pDONR222.1 and sequenced. Under the direction of the CaM35S, the coding sequence of pMDC32 was cloned by the Gateway system (Invitrogen, Waltham, MA, USA), and 35S: *PeERF1* and 35S: SRDX-*PeERF1* plant expression vectors were obtained. *P. alba × P. glandulosa* were genetically transformed using the callus transformation technique. More than 15 transgenic lines were developed after screening on media containing 50 mg/L hygromycin (Invitrogen, Waltham, MA, USA). Finally, three distinct transgenic lines with high levels of candidate genes were employed in further investigations. 

### 4.8. Subcellular Localization and Self-Activation Experiment of PeERF1

The coding region of *PeERF1* lacking a termination codon was fused to the 5’terminus of the coding region of GFP to form *PeERF1*-GFP fusion protein. The proper coding sequence was cloned into pEarley Gate 101(C-YFP) under the control of the CaM35S utilizing the Gateway system (Invitrogen, Waltham, MA, USA), and cloned into pDONR222.1 for sequencing. The primers are shown in Supplementary Table S8. Agrobacterium tumefaciens with fusion vectors *35S: PeERF1-YFP* was immersed in tobacco leaves by injection and cultured in darkness at room temperature for 36 h. The epidermises were then stained in the dark for 15 min with 100 ng/mL DAPI. The epidermis was dyed, rinsed three times with phosphate-buffered saline, and the fluorescence was analyzed using a confocal laser scanning microscope (LSM 700, Zeiss, Germany).

To verify whether *PeERF1* has self-activation activity, we carried out a yeast two-hybrid test. we used the Yeast maker™ Yeast Transformation System 2 (Takara, Dalian, China) and performed yeast transformation according to the instructions in the user manual. The effector vector and reporter vector were tested in the Y2H Gold yeast strain using the SD/-Trp solid medium. As a negative control, pGBKT7-Rec2 transformed yeast cells were used. To examine the interactions, the successfully transformed yeast cells were cultured in SD/-Trp liquid medium and then coated on SD/-Trp and SD/-Trp and SD/-Trp/X-a-gal (20 mM) solid medium.

### 4.9. Promoter Assay of PeERF1

To investigate the tissue localization of *PeERF1* gene expression, we transformed *P. alba × P. glandulosa* using PMDC164 plasmid vector containing the *PeERF1* promoter. We use about 1524 bp upstream sequence of *PeERF1* gene in *P. euphratica* genome for primer design. The primers are in Supplementary Table S8, and the Gateway method was used to construct the GUS promoter gene reporter vector. Transgenic callus of poplar was produced using the previously published Agrobacterium-mediated technique. The activity of the GUS enzyme was examined using a type microscope.

### 4.10. One-Hybrid Yeast Experiment

To verify whether PeERF1 can bind to the GCC-box (AGCCGCC), DRE motif (TACCGACAT), TTG1 motif (TTGTTTTGTT), TTG2 motif (TTTTTTTGTT), and their mutant elements GCC-m1 (AGTTGCC), GCC-m2 (ATCCTCC), GCC-m3 (TTTTTTTT), DRE-m1 (TATTGACAT), DRE-m2 (TACCTTCAT), TTG1-m1 (TTGCCTTGTT), TTG1-m2 (TTGTTTTGCC), TTG2-m1 (TTTTTCCGTT), and TTG2-m2 (TTTTTTTGCC), we performed yeast one-hybrid assay. The core sequences of the above four components and their corresponding base mutation sequences were concatenated in three repeats, and EcoR I and Sac I restriction sites were introduced into the upstream and downstream of each concatenated fragment to design primers. We performed yeast transformation using the Yeast maker™ (Takara, Dalian, China). The effector and reporter vectors were introduced into the Y187 yeast strain and tested on DDO solid medium. The pHIS2-p53/pGADT7-Rec2-p53 as positive control and pHIS2-p53/pGADT7Rec2-*PeERF1* as negative control co-transformed yeast cells were utilized. To examine the interactions, the successfully transformed yeast cells were grown in DDO liquid medium and then coated on DDO and TDO/3-AT (75 mM) solid medium, and then tested for spots.

### 4.11. Analysis of Physiological Characteristics of Transgenic P. alba × P. glandulosa Plants

On 1/2 MS media with 0, 50, 75, and 100 mM NaCl, transgenic and wild type (WT) were cultivated. The root length, plant height, and fresh weight of plants at one month old were measured using ten biological duplicates. In the greenhouse, one-month-old WT and transgenic plants were placed in soil containers for a month. They were subsequently given a treatment of 150 mM NaCl for 7 days, with water as a control. We assessed the POD, SOD, MDA, electrolyte leakage rate (LR), and leaf chlorophyll content (CC) after the stress treatments. The LR was determined using a DDSJ-308A conductivity meter and following the manufacturer’s recommendations. According to Li’s approach, the CC of several plants was calculated [29]. The activities of SOD, POD, MDA, and Soluble sugar content were measured by following the manufacturer’s instructions (Beijing Solarbio Technology Co., Ltd., Beijing, China). Kumar and Huang’s approach was used for staining of histochemical using 3,3′-diaminobenzidine (DAB) and Nitrotetrazolium blue chloride (NBT) [65].

### 4.12. Gene Expression Characterization Using RNA-Seq

We selected WT and *PeERF1* transgenic plants and transplanted them into potted plants containing peat soil and vermiculite (3:1). After growing for one month, salt stress was performed for 0 h and 24 h. The top leaves of plants were taken and stored at −80 °C. Three biological replicates were performed on the samples (the specific numbers were C-WT-1/2/3 for 0 h treatment of wild type, C-OE-1/2/3 for 0 h treatment of overexpression of 35S: *PeERF1*, C-SE-1/2/3 for 0 h treatment of 35S: SRDX-*PeERF1*, T-WT-1/2/3 represented 24 h treatment of wild type, T-OE-1/2/3 represented 0 h treatment of overexpression of 35S: *PeERF1* and T-SE-1/2/3 represented 0 h treatment of dominant expression of 35S: SRDX-*PeERF1*).

Total RNA was extracted using a Trizol reagent kit (Invitrogen, Carlsbad, CA, USA) according to the manufacturer’s protocol. RNA quality was assessed on an Agilent 2100 Bioanalyzer (Agilent Technologies, Palo Alto, CA, USA). Eukaryotic mRNA was enriched by Oligo(dT) beads (Epicentre, Madison, WI, USA). The purified double-stranded cDNA fragments were prepared using the NEBNext Ultra RNA Library Prep Kit for Illumina (NEB #7530, New England Biolabs, Ipswich, MA, USA), which included end repair, A base addition, and ligation to Illumina sequencing adapters. The AMPure XP Beads (1.0×) were used to purify the ligation process. Ligated fragments were size-selected using agarose gel electrophoresis and amplified using polymerase chain reaction (PCR). Gene Denovo Biotechnology Co. sequenced the generated cDNA library on an Illumina Novaseq6000 (Guangzhou, China).

The short read alignment tool Bowtie2 [59,66] (version 2.2.8) was used to map reads to the ribosomal RNA (rRNA) database. A reference genome index was constructed, and paired-end clean reads were mapped to it with HISAT2.2.4 [57] with the “-rna-strandness RF” and other parameters set to default. StringTie v1.3.1 was used to create the mapped reads of each sample in a reference-based technique [67,68]. For each transcription area, an FPKM value was computed using the RSEM program to assess the amount and variability of its expression [69].

## 5. Conclusions

In this study, we obtained the DEGs in *P. euphratica* leaves with different salt treatments, which were involved in biological processes and pathways, and screened eight potential genes based on the co-expression network and GWAS results. Under salt stress, we found the accumulation of osmotic adjustment chemicals and an increase in antioxidant enzyme activity in *P. euphratica*. Many DEGs were shown to be enriched in stress-related activities such as transcriptional regulation, stress response, cell death, and so on. *PeERF1* has been shown to improve salt tolerance in transgenic plants by overexpression and dominant inhibitory expression in *P. alba* × *P. glandulosa*. Our results will contribute to resolve the salt tolerance mechanism of *P. euphratica*, help to identify and breed poplar salt tolerance candidate genes, provide material for future related studies, and also lay the foundations for plant stress tolerance.

## Supplementary Materials

The following supporting information can be downloaded at: https://www.mdpi.com/article/10.3390/ijms23073727/s1.
