# Supplementary figures and images for: Transcriptome Analysis of Populus euphratica under Salt Treatment and PeERF1 Gene Enhances Salt Tolerance in Transgenic Populus alba × Populus glandulosa
Source: Int J Mol Sci. 2022 Mar 28;23(7):3727. doi: 10.3390/ijms23073727 (PMC8998595; doi:10.3390/ijms23073727)

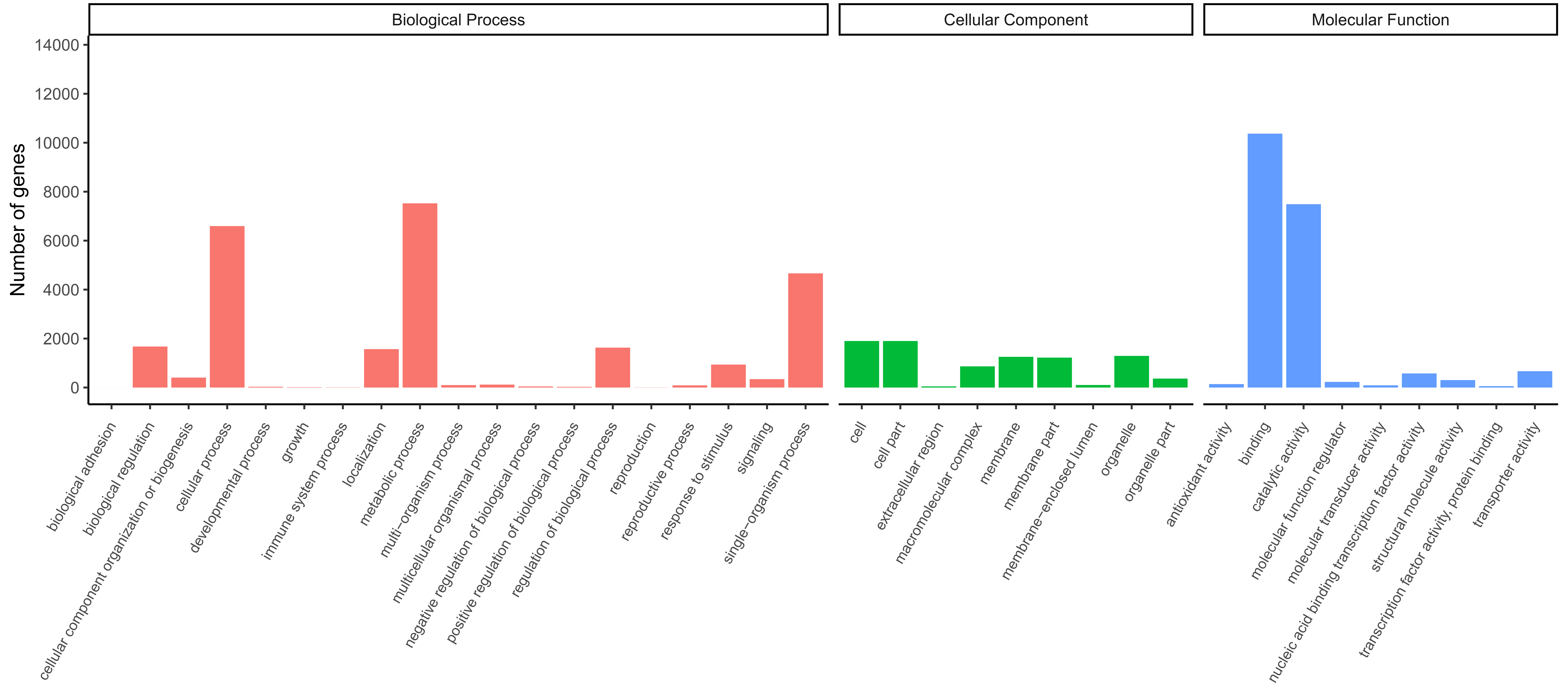

Supplement: Supplementary file 1 [file ijms-23-03727-s001.zip › Figure S1.jpg]

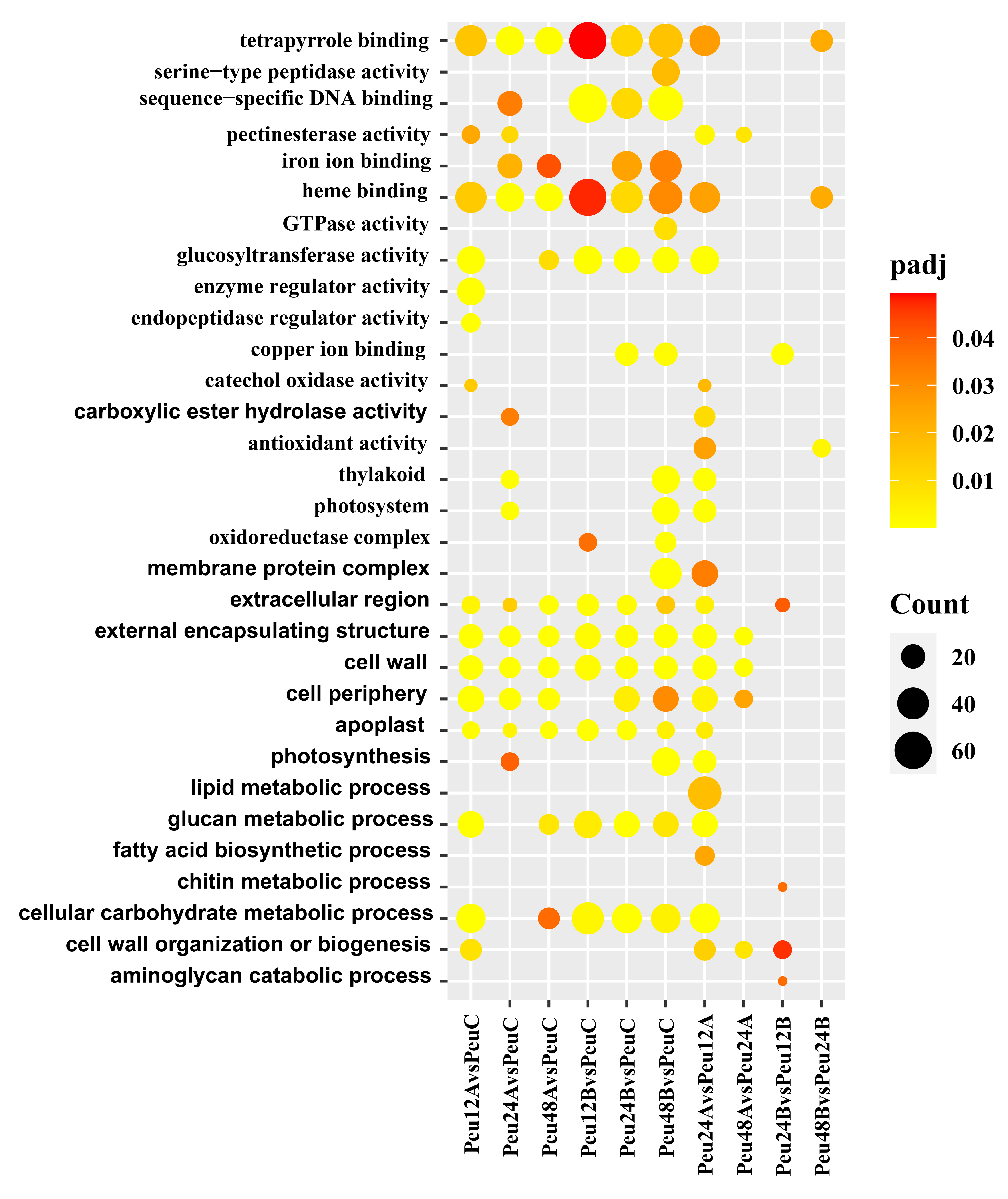

Supplement: Supplementary file 1 [file ijms-23-03727-s001.zip › Figure S2.jpg]

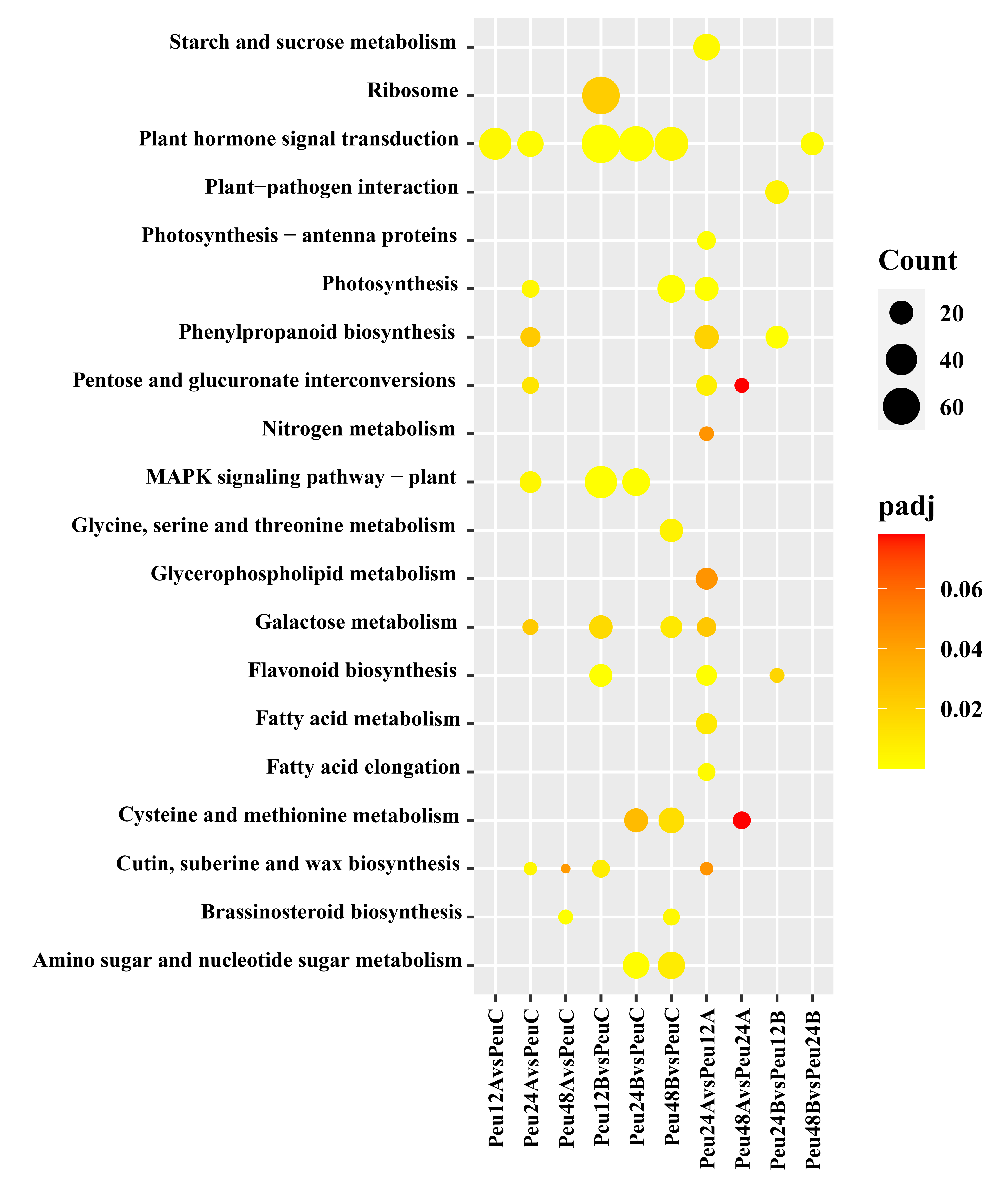

Supplement: Supplementary file 1 [file ijms-23-03727-s001.zip › Figure S3.jpg]

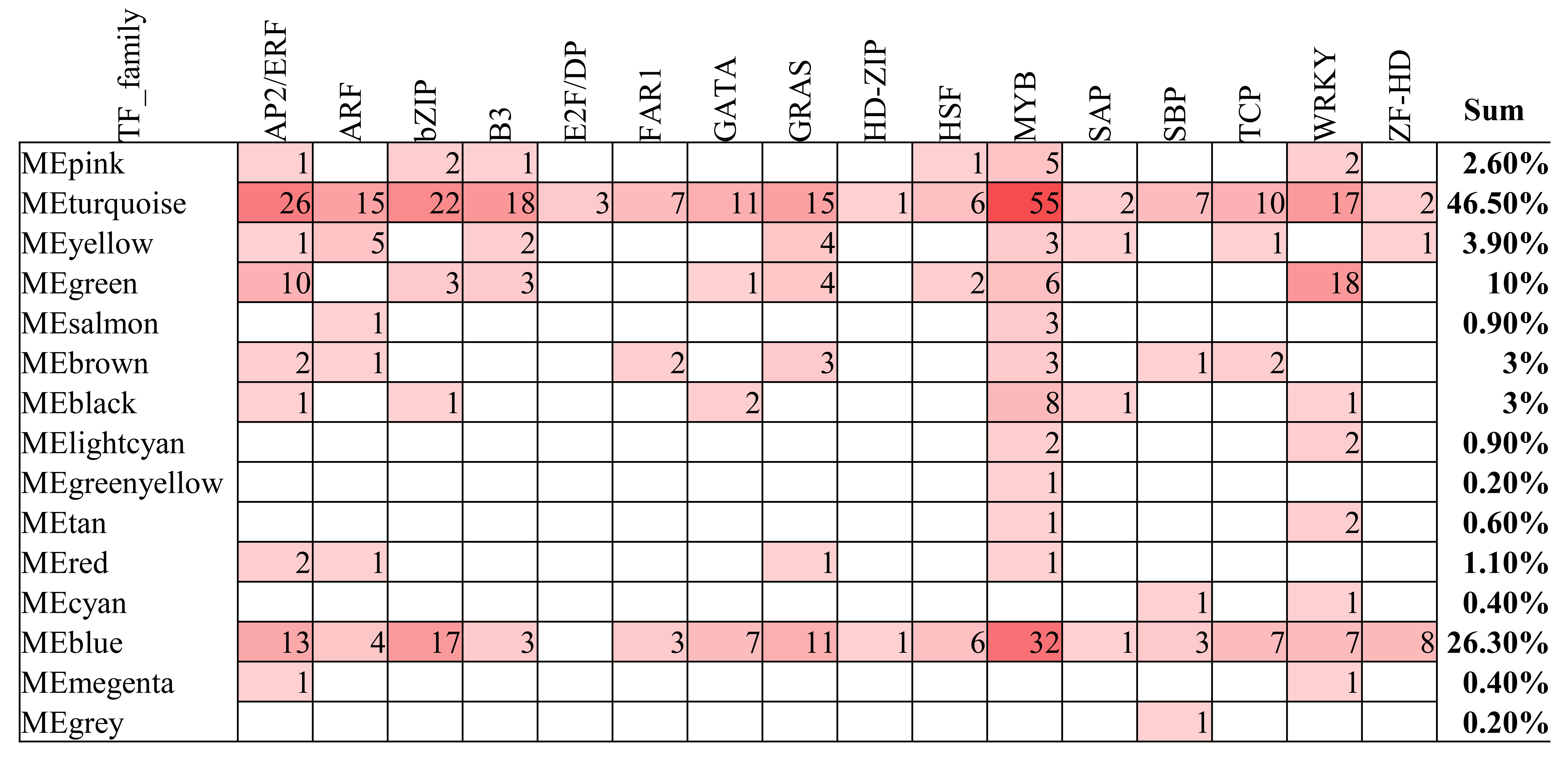

Supplement: Supplementary file 1 [file ijms-23-03727-s001.zip › Figure S4.jpg]

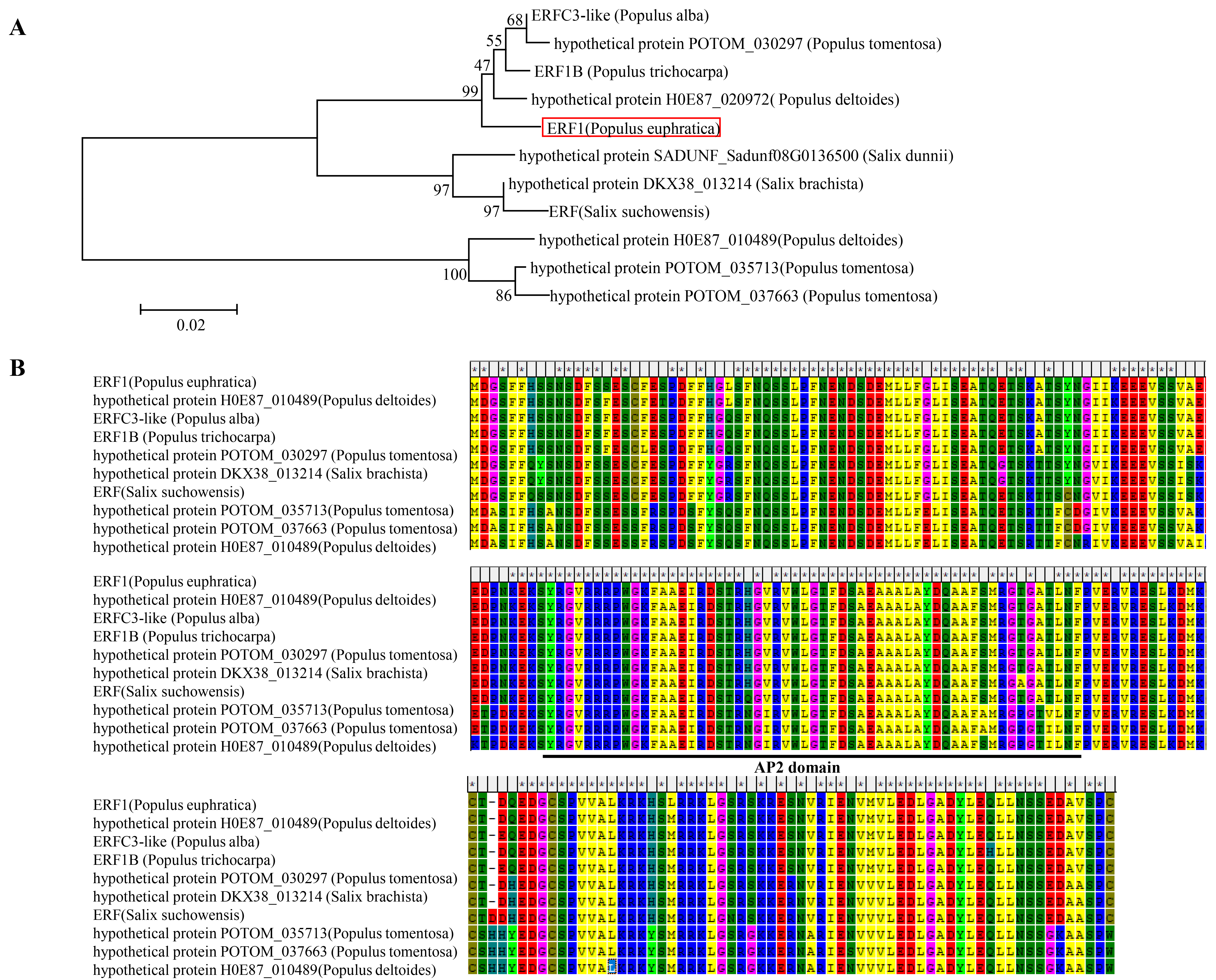

Supplement: Supplementary file 1 [file ijms-23-03727-s001.zip › Figure S5.jpg]

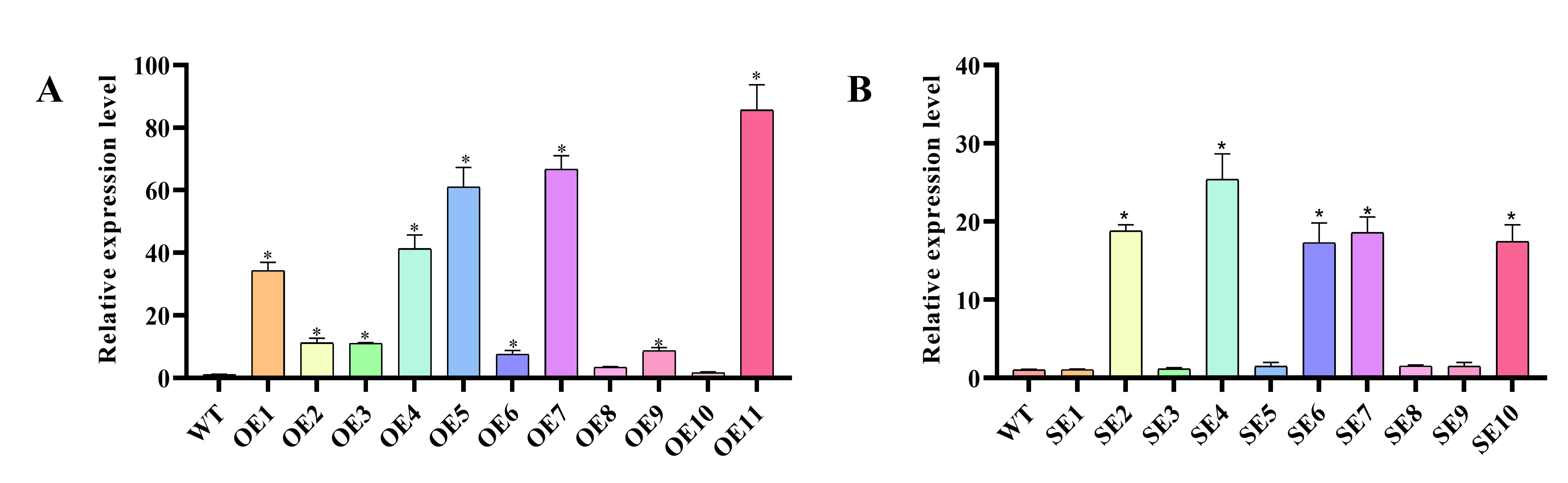

Supplement: Supplementary file 1 [file ijms-23-03727-s001.zip › Figure S6.jpg]

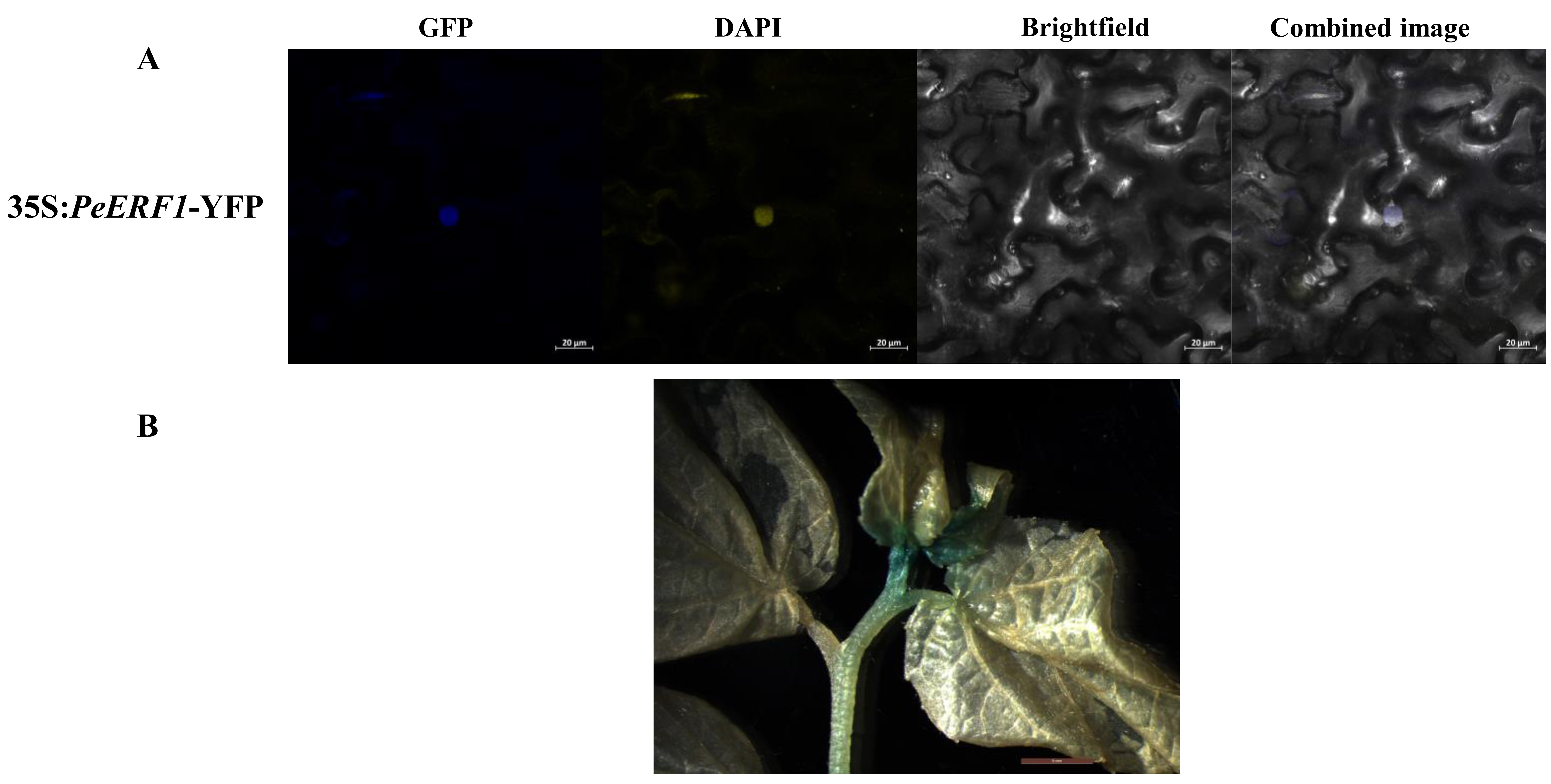

Supplement: Supplementary file 1 [file ijms-23-03727-s001.zip › Figure S7.jpg]
